# MR Image-Based Attenuation Correction of Brain PET Imaging: Review of Literature on Machine Learning Approaches for Segmentation

**DOI:** 10.1007/s10278-020-00361-x

**Published:** 2020-06-30

**Authors:** Imene Mecheter, Lejla Alic, Maysam Abbod, Abbes Amira, Jim Ji

**Affiliations:** 1 Department of Electronic and Computer Engineering, Brunel University London, Uxbridge, UK; 2 Department of Electrical and Computer Engineering, Texas A & M University at Qatar, Doha, Qatar; 3 Magnetic Detection and Imaging Group, Faculty of Science and Technology, University of Twente, Enschede, Netherlands; 4 Institute of Artificial Intelligence, De Montfort University, Leicester, UK; 5 Department of Electrical and Computer Engineering, Texas A & M University, College Station, TX USA

**Keywords:** MR image-based attenuation correction, Image segmentation, Machine learning, Deep learning, PET/MR

## Abstract

Recent emerging hybrid technology of positron emission tomography/magnetic resonance (PET/MR) imaging has generated a great need for an accurate MR image-based PET attenuation correction. MR image segmentation, as a robust and simple method for PET attenuation correction, has been clinically adopted in commercial PET/MR scanners. The general approach in this method is to segment the MR image into different tissue types, each assigned an attenuation constant as in an X-ray CT image. Machine learning techniques such as clustering, classification and deep networks are extensively used for brain MR image segmentation. However, only limited work has been reported on using deep learning in brain PET attenuation correction. In addition, there is a lack of clinical evaluation of machine learning methods in this application. The aim of this review is to study the use of machine learning methods for MR image segmentation and its application in attenuation correction for PET brain imaging. Furthermore, challenges and future opportunities in MR image-based PET attenuation correction are discussed.

## Introduction

**Fig. 1:**
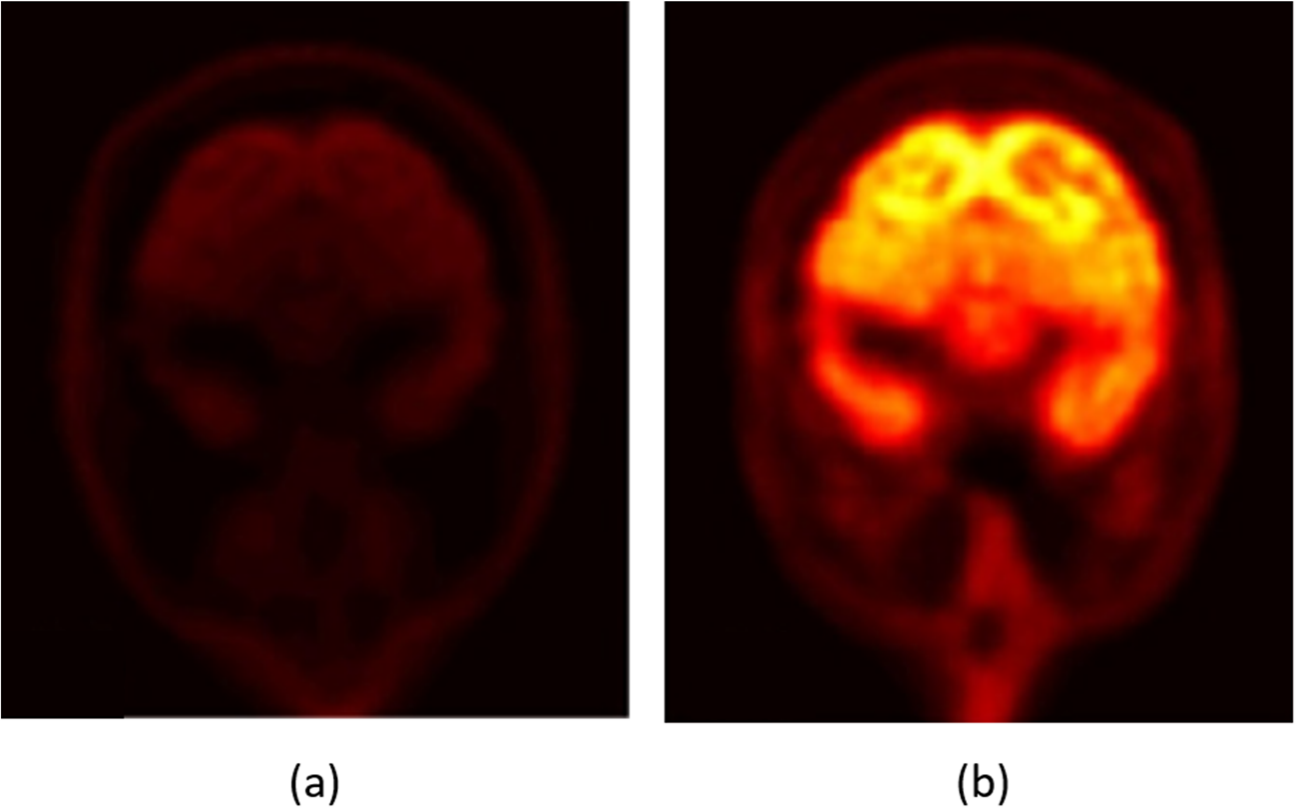
A reconstructed PET image without attenuation correction (**a**), and with attenuation correction (**b**) using the [(18)F]-fluorodeoxyglucose ((18)F-FDG) radiotracer. Adopted form [4]

Positron emission tomography (PET) is an imaging modality that provides direct imaging of physiological biomarkers using radiolabeled gamma-ray emitting molecules. The knowledge of the tissue-dependent attenuation map, needed for attenuation correction, is a critical step to achieve an accurate PET image reconstruction. Figure 1 shows the effect of attenuation correction on reconstructed PET images. The attenuation map is usually obtained by performing an additional scan using X-ray computed tomography (CT) [1]. CT image intensity measured in the Hounsfield unit is a map of the normalized X-ray attenuation coefficients, which reflects the anatomical, physiological and pathological states of the underlying tissues. Therefore, the CT image can be mathematically converted to the equivalent linear X-ray attenuation coefficients [2, 3]. Since X-rays and gamma-rays have similar attenuations in biological tissues, X-ray CT is the most straightforward way for PET attenuation correction. However, it introduces additional ionizing radiations to the imaging subjects.

On the other hand, magnetic resonance (MR) imaging is nowadays considered the premier modality for imaging the brain structures and functions due to its excellent soft tissue contrast, high spatial resolution, and lack of ionizing radiation. Therefore, MR images have been extensively used for visualizing, analyzing, diagnosis, treatment planning, and follow-up of a variety of neurological conditions.

To take advantages of both MRI and PET, hybrid PET/MR systems were recently introduced and applied in the clinical molecular imaging applications [5]. However signal intensity in MR images is not directly correlated to attenuation coefficient which is required for attenuation correction in PET image reconstructions [2]. Therefore, MR image-based attenuation correction has become one of the challenges in PET/MR systems [6]. There are different approaches are used to addressing this challenge which are discussed in “MR Image-Based Attenuation Correction for Brain PET Imaging”. T1-weighted and T2-weighted MR images are commonly used in MR image-based attenuation correction. Other MR image pulse sequences which provide more details in morphological information can also be used for this purpose. These include diffusion-weighted images (DWI) [7], short echo time (STE) [8], ultra-short echo time (UTE) [9], zero echo time (ZTE) [10], dynamic contrast-enhanced (DCE) imaging [11], and magnetization-prepared rapid acquisition gradient echo (MP-RAGE) sequences [12].

Current research utilizes different machine learning techniques and MR image data acquisition sequences to perform MR image segmentation for different medical applications, including PET/MR attenuation correction. DWI sequence [13] or a combination of sequences [14, 15] is the most routinely used for the diagnosis and follow-up in ischemic and hemorrhagic stroke. For quantitative analysis in multiple sclerosis, T2-weighted MR images is commonly used sequence either as a single imaging sequence [16] or in a multi-sequence approach [17–19]. T1-weighted MR images are frequently used to assess biomarkers of Alzheimer’s disease such as hippocampal atrophy, ventricle enlargement and cortex shrinkage [20, 21]. Brain tumor segmentation of MR images received much attention over the last decade, especially for treatment planning and follow-up. A range of MR image sequences were used as input to segmentation procedure: single MR image sequence with [22] and without [23] contrast agent, or multi-sequence MR images with [24–27] or without contrast [24, 28].

The process of segmentation is performed by segmenting the brain MR images into three main tissue classes: white matter (WM), gray matter (GM), and cerebrospinal fluid (CSF) as well as the lesion regions. These have been a large body of literatures on brain image segmentation methods as thresholding, edge based, watershed based, and various machine learning techniques. For instance, random forest classifier has been used for diagnosis of Alzheimer disease by biomarkers such as segmenting white matter lesions [29] or hippocampus for diagnosis [30], and for segmentation of brain tumor lesions [31]. Support vector machine (SVM) showed good segmentation results in white matter lesion [32], multiple sclerosis region [33], brain tumor lesions [24] or to diagnose Alzheimer disease [20]. Additionally, probabilistic models were proposed to efficiently segment the brain tissue into three classes or to only extract the region of interest by applying the inverted Dirichlet mixture Model [34], Markov random field [35] and Bayesian model [36]. Furthermore, neural network such as multilayer perceptron [37], self-organizing map (SOM) network [38], extreme learning machine (ELM) [39], and cellular neural network [40] achieved also good results in segmenting the brain into different regions. Unsupervised learning, specifically clustering techniques, have been widely used for skull striping [41], white matter lesion segmentation [42], and tumor region segmentation [23]. Recently, deep learning proved powerful performance in several brain segmentation applications using convolutional neural networks (CNN) for skull stripping [43], stroke lesion segmentation [44], multiple sclerosis segmentation [45], and brain tumor segmentation [27].

The aim of this literature review is to highlight the recent progress made on MR image-based PET attenuation correction using machine learning for segmentation of brain tissues. The structure of this review is as follows: Section “MR Image-Based Attenuation Correction for Brain PET Imaging” introduces the MR image-based attenuation correction for brain PET imaging. Section “Machine Learning-Based Segmentation Methods for PET Attenuation Correction in Brain Imaging” represents the machine learning-based segmentation methods for PET attenuation correction in brain imaging. Section “Performance Metrics and Clinical Evaluation” discusses the evaluation metrics and clinical evaluation. The challenges and opportunities are discussed in “Challenges and Future Opportunities” and the conclusion is presented in “Conclusion”.

## MR Image-Based Attenuation Correction for Brain PET Imaging

**Fig. 2:**
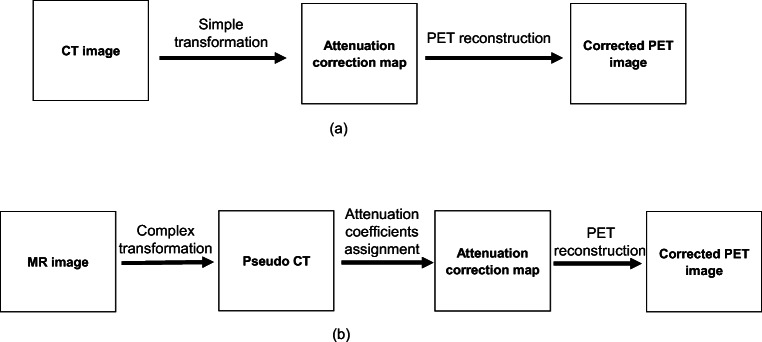
The overview process of deriving attenuation correction maps from : **a** CT images, **b** MR images

With the recent introduction of hybrid PET/MR scanners, PET attenuation correction maps need to be generated from MR images. Unlike CT, the MR image signal intensity has no direct mapping to PET attenuation coefficients. For instance, bone and air have similar intensity values in conventional MR images while they have quite different attenuation coefficients [3]. Figure 2 illustrates the difference between CT imaging-based and MR imaging-based attenuation correction.

Generating pseudo CT from MR images requires segmented MR images where tissue classes assigned a specific attenuation coefficient. Moreover, clinically adopted method for MR image-based attenuation correction, also available for commercial PET/MR scanners, involves MR images segmentation as a basis to generate pseudo CT images [46]. MR images are commonly segmented into three or more tissue classes then predefined attenuation coefficients will be assigned to each voxel tissue class. Hence, there is a great need for a reliable method to generate the attenuation correction coefficients from MR images. As shown, it requires complex transformation to produce pseudo CT from MR images. The main complex transformations to map a MR image to a pseudo CT images are as follows: segmentation, atlas, and machine learning which are discussed in the following paragraphs.

The segmentation methods are traditionally considered the most robust and simple methods adopted in clinical domain for MR image-based attenuation correction for PET images [47–49]. The first clinical hybrid PET/MR system uses the two-point Dixon gradient echo sequence which simplifies the segmentation of MR images into different tissue classes [4]. Nowadays, the commercial PET/MR systems segment images into three or four tissue classes [50], with the voxels of each tissue class assigned an approximated predefined linear attenuation coefficient [51] producing the attenuation correction map. MR image segmentation was performed using different approaches starting with simple techniques such as level set [52, 53], thresholding [50, 54–60], and radon transform [59] until more complicated techniques such as clustering [61–63], classification [64] and deep learning [65–67]. Table 1 illustrates different segmentation methods applied to different MR image sequences. The main challenge of segmentation is the accurate delineation of bone tissue [47]. Furthermore, there is also a disagreement on the value of a linear attenuation coefficient to be assigned for the bone tissue [54].

**Table 1:** The different segmentation methods applied on different MR sequences

Reference	Segmentation technique	MR sequence
[52]	Level set	STE
[53]	Level set	UTE
[55]	Thresholding	UTE
[57]	Thresholding	ZTE
[58]	Thresholding	Dixon
[59]	Radon transform	T1 weighted
[62]	Clustering	STE and Dixon
[63]	Clustering	T1 weighted
[64]	Classification	DCE, MP-RAGE, T1 weighted
[73]	Classification	Dixon
[65]	Deep learning	T1 weighted
[66]	Deep learning	UTE and out-of-phase echo
[84]	Deep learning	T1 weighted

The atlas-based methods, also referred to as the registration-based methods, involve image registration between atlas/template images (MR/CT image pairs) and the target MR image using nonlinear transformation. First, the MR image of the subject is co-registered with the atlas MR image. Then, the obtained transformation is applied to atlas CT image to create subject specific attenuation correction map [48]. The quality of PET reconstruction is highly dependent on the registration algorithms accuracy. Different atlas-based techniques were proposed in the literature [46, 68–70]. Most of the atlas-based methods use machine learning to estimate the pseudo CT image using MR image features such as signal intensity and geometric metrics to learn the relationship between MR signal and Hounsfield units in CT. This method is time consuming and potentially have decreased accuracy under anatomical variations, especially in cases undergoing neurosurgery [71].

Machine learning methods are related to both segmentation- and atlas-based methods where different algorithms are applied either to perform MR image segmentation [61–64, 72, 73] or post-registration process to learn the complex mapping from MR images to CT in order to generate the pseudo CT images [74–83]. Different machine learning techniques have been applied such as Gaussian mixture regression model, *k*-nearest neighbors (kNN) regression, random forest classifier, neural networks, clustering techniques, and deep learning. In the next section, MR images segmentation methods using machine learning for brain PET attenuation correction are reviewed in detail. These include image clustering, image classification, and deep learning.

## Machine Learning-Based Segmentation Methods for PET Attenuation Correction in Brain Imaging

Tables 2 and 3 summarize the three main categories of machine learning techniques proposed for MR images segmentation: clustering, classification, and deep learning.

**Table 2:** Segmentation-based MR attenuation correction methods for brain imaging

Reference	Segmentation technique	MR sequence	Ground truth	Evaluation metrics
[8]	Fuzzy C-means clustering morphologic operations	STE and Dixon	CT-based attenuation correction	Visual comparison Accuracy (cortical bone) = 0.96 Specificity (cortical bone) = 0.97 Sensitivity (cortical bone) = 0.75 Correlation coefficient (*R*^2^) > 0.95
[62]	Fuzzy C-means clustering	STE and Dixon	Manual segmented MRI	Accuracy (bone) = 0.98 ± 0.01 Sensitivity (bone) = 0.93 ± 0.02 Specificity (bone) = 0.98 ± 0.01
[63]	Fuzzy C-means clustering	T1-weighted	TX-based attenuation correction	Dice (whole image) = 85.2 ± 2.6*%* Average relative difference = 4.2*%* PSNR = 44.5 ± 6.9
[72]	Fuzzy C-means clustering	Undersampled UTE and mDixon	CT-based attenuation correction	Coordinates of different tissue classes CT histogram curve MAPD = 130 ± 16 HU Mean prediction deviation = − 22 ± 29 HU
[4]	SVM	UTE and T1-weighted	CT-based attenuation correction	Dice = 70 ± 34*%* Voxel-wise error = 27.8 ± 8.3*%* Region-wise error = 49.7 ± 6.2*%*
[61]	Probabilistic Neural Network	UTE	CT-based attenuation correction	Visual comparison Accuracy = 92*%* Dice (whole head)= 0.66± 0.07
[64]	Random forest classifier	DCE, T1-weighted, and MP-RAGE sequences	CT image	Visual comparison Dice (air) = 0.83 ± 0.06 Dice (bone) = 0.98 ± 0.01 Accuracy (bone) = 0.96 ± 0.02 AUC = 0.9875 ± 0.0002
[73]	Adaptive boosting classifier	T1 weighted	CT-based attenuation correction	Whole-brain SUV estimation bias = 95*%*
[65]	Deep learning: convolutional encoder-decoder	T1 weighted	CT-based attenuation correction	Dice (air) = 0.971 ± 0.005 Dice (soft tissue) = 0.936 ± 0.011 Dice (bone) = 0.803 ± 0.021 PET reconstruction error = − 0.7 ± 1.1

**Table 3:** Segmentation-based MR attenuation correction methods for brain imaging (Continued)

Reference	Segmentation technique	MR sequence	Ground truth	Evaluation metrics
[66]	Deep learning: convolutional encoder-decoder	UTE and out of phase	CT-based attenuation correction	Dice (air) = 0.76 ± 0.03 Dice (soft tissue) = 0.96 ± 0.006 Dice (bone) = 0.88 ± 0.01 Relative PET error = < 1*%*
[84]	Deep learning: generative adversarial network	T1 weighted	CT-based attenuation correction	Dice (bone) = 0.77 ± 0.07 Relative volume difference (bone) = 45.2 ± 20.1 Mean absolute surface distance (bone) = 2.4 ± 0.65 Mean error = − 46 ± 150 Mean absolute error = 302 ± 79 PET error (bone) = 1.2 ± 13.8 PET error (soft tissue and air) = 3.2 ± 13.6 RMSE (PET) = 168 ± 52 PSNR (PET) = 28.43 ± 1.16 SSIM (PET) = 0.87 ± 0.04

### Clustering

**Fig. 3:**
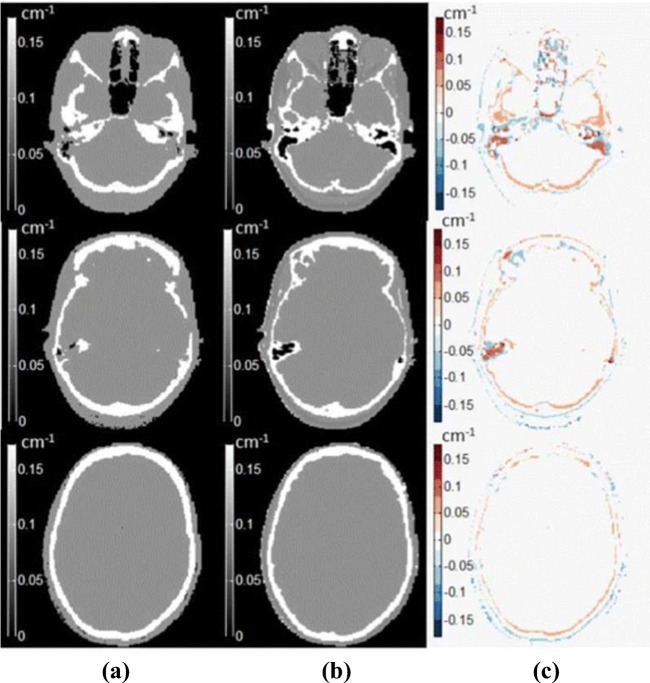
MR image segmentation results achieved by [8] using clustering technique with **a** the reference CT images, **b** the segmented MR images, and **c** the difference between the two modalities

Khateri et al. [62] used a combination of STE sequences with 2-point Dixon technique along with a fuzzy C-means (FCM) clustering-based segmentation method to detect bone tissue. They segmented the brain into four clusters, namely cortical bone, soft tissue, adipose tissue, and air. They concluded that the clustering technique is an appropriate approach to segment bone and air in the sinusoidal area. The bone segmentation results achieved more than 90% in terms of accuracy, sensitivity, and specificity. However, the eye area can be misclassified as bone. The results were validated using manually segmented bone regions on STE MR images by a neuroradiologist expert. Later, the same team (Khateri et al. [8]) applied FCM clustering to segment the brain into three tissue classes (cortical bone, soft tissue, and air) using the same combination of MR sequences (STE + Dixon). They used morphologic operations as post segmentation to reduce susceptibility error. This method was evaluated with CT-based attenuation correction maps as shown in Fig. 3. The visual comparison showed the high similarity between MR and CT segmentation results. The evaluation measures are signal-to-noise ratio (SNR), accuracy, sensitivity, specificity, and correlation plots. The segmentation results proved that the combination of STE sequence with a clustering technique is a potential alternative for UTE sequences for PET attenuation correction. They concluded from the obtained attenuation correction maps that the ethmoid sinuses are the most error-prone areas with the largest difference in the paranasal area.

Fei et al. [63] developed a multiscale segmentation approach using radon transform of T1-weighted MR images to segment the head image into skull, scalp, and brain tissue. Then, the brain tissue was classified into three classes: GM, WM, and CS by applying unsupervised clustering technique. The images were firstly processed with anisotropic diffusion filter to construct a multiscale image series in order to overcome the blurred edges drawback. Then, a multiscale FCM technique was applied to allow multiscale processing from the coarse to fine levels. Afterwards, predefined attenuation coefficients were assigned to each tissue class. The segmentation is evaluated using dice similarity measurement. The quality of PET images is compared with transmission (TX)-based attenuation correction by visual inspection followed by quantitative measurements which are relative difference, mean squared error (MSE), and peak signal-to-noise ratio (PSNR). The overlap ratio between the segmented and ground truth CT is around 85% and the difference between the MR- and TX-based attenuation correction maps is less than 7%.

Su et al. [72] proposed an attenuation correction method using a single acquisition, undersampled UTE-mDixon, MR images. They applied FCM clustering algorithm to segment the head into five different tissue classes including brain, air, fat, fluid, and bone. After optimizing MR images, three image features which are Dixon-fat, Dixon-water, and *R*^2^ were used as input to the unsupervised clustering algorithm. The segmented MR voxels were assigned attenuation correction coefficients to generate the pseudo CT images. The coordinates of the centroids of different tissue types were calculated to evaluate the segmentation results. Then, the obtained pseudo CT images are compared with measured low-dose CT images visually and subjectively by calculating the CT histogram and mean absolute predication deviation (MAPD).

### Classification

Shi et al. [4] proposed bone refinement method for existing attenuation correction map. This method started with an existing attenuation correction map from the vendor or obtained from any segmentation-based method then refined the attenuation correction map gradually by learning the relationship between the MR image and the attenuation correction map. The learning process performed using multiresolution regional learning approach by applying SVM classifier to refine the attenuation correction coefficients using training features of UTE1, UTE2, and MP-RAGE sequences. The resulting attenuation correction map was compared with vendor map and CT-based attenuation correction map by measuring the bone recovery rate, dice coefficient, voxel-wise error, and region-wise error. The measurements showed that the proposed method enhanced the attenuation coefficient map. The reconstructed PET images displayed that the error is larger in the regions near the skull. They also concluded from the visual inspection that the proposed method reduced the underestimation of PET activities. However, the results are still not comparable with atlas-based methods.

Santos et al. [61] performed a learning-based segmentation method of the skull using probabilistic feed-forward neural network which requires user interaction. UTE sequences were used to segment the brain into four classes: air, brain + soft tissue, CSF, and bone. The model was trained using two patches of raw MR intensities (UTE1 and UTE2) as input features. This method was compared with CT-based attenuation correction map by calculating the accuracy, the dice similarity score, and the visual comparison. They found that this approach achieved high values for the co-classification of air and soft tissue. However, the bone values are low. This algorithm depends on the patient’s MR intensity values which affects the performance if there is a big difference between patients’ MR intensities.

**Fig. 4:**
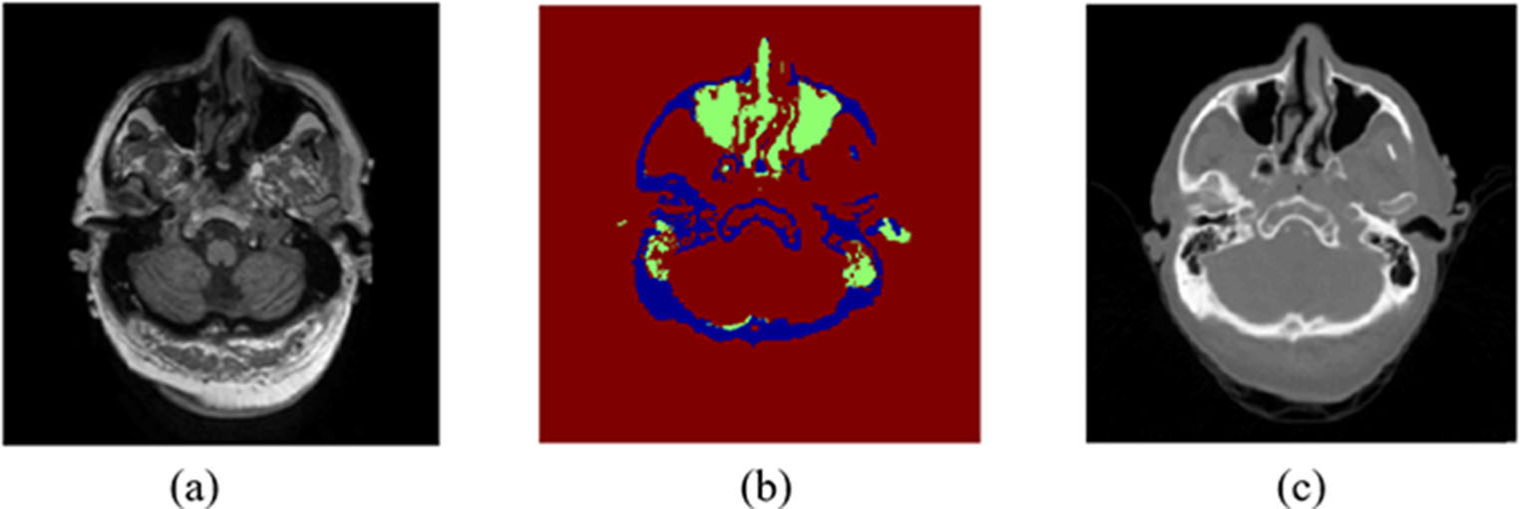
MR image segmentation result achieved by [64] using voxel classification to differentiate bone from air. **a** T1-weighted MR image, **b** segmented MR image, and **c** corresponding CT image as ground truth

Chan et al. [64] proposed a segmentation method based on tissue classification to differentiate bone from air. Brieman’s random forest classifier was trained using a set of features include gradient, textural, and contextual features extracted from MP-RAGE MR sequences and uncorrected PET images. The segmentation results of MP-RAGE images are shown in Fig. 4. Dice similarity score for each tissue class, accuracy, area under the curve (AUC) of the receiver operating characteristic (ROC) curve, and visual assessment were used to evaluate the segmentation performance. The evaluation metrics were compared with CT-based classification as ground truth. They concluded that the segmentation results were improved when including the uncorrected PET features compared with segmentation using MR image features only.

Koesters et al. [73] applied Adaboost classifier to extract bone tissue from T1-weighted MR sequences. Afterwards, the bone attenuation coefficient was added to Dixon-based attenuation map from the manufacturer that reflects four tissue attenuation coefficients but not bone tissue. This method was evaluated by comparing the standardized uptake value (SUV) estimation between CT, Dixon, and the proposed model for whole-brain and regional analyses. The results showed that there is a significant improvement in terms of SUV estimation bias by comparing the proposed model with Dixon-based MR images. The proposed approach reduced the whole brain SUV estimation bias of Dixon-based approach by 95% and a similar residual SUV bias to CT-based approach.

### Deep Learning

Deep learning is an emerging technology in machine learning which represents advanced and more complex forms of neural networks. Deep networks are self-learning structures capable of learning high-level image features and modeling a nonlinear mapping between different image spaces through the convolution process. These methodologies showed their superiority in several medical applications, which paved the way for their recent exploration for PET attenuation correction by generating attenuation maps using different MR sequences.

There are only few publications that applied deep learning to brain MR image segmentation for PET attenuation corrections [65–67]. Each of these proposed studies used different MR image sequences and network architecture to train the deep network.

**Fig. 5:**
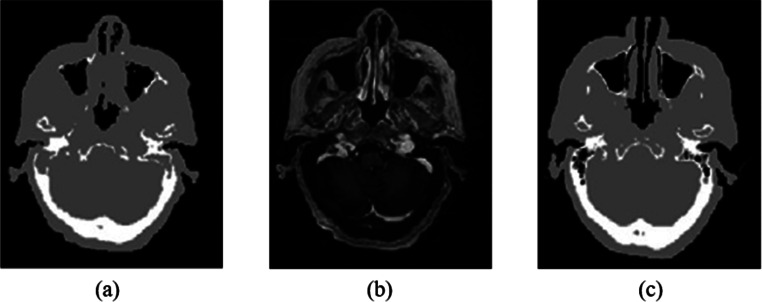
**a** Pseudo CT image obtained by segmenting **b** T1-weighted MR image with the use of **c** CT image as a ground truth [65]

Liu et al. [65] applied a deep convolutional encoder-decoder network called Segnet [85] using T1-weighted MR images. This work required a co-registration between CT and MR images before the training process and the creation of ground truth. To train the network, the labels were generated by segmenting the CT images into three classes (air, soft tissue, and bone) using intensity-based thresholding technique. These classes were assigned an attenuation coefficient value to generate the pseudo CT images as illustrated in Fig. 5. Dice similarity score for each class was calculated to evaluate the segmentation results and PET reconstruction error was measured to quantify the obtained PET image. The proposed method was compared with Dixon-based soft tissue and air segmentation and anatomic CT-based template registration. The results achieved accurate pseudo CT scans and good PET images with lower errors compared with Dixon-based and CT-based attenuation correction. The main limitation of this approach is the intrasubject registration which would affect the segmentation performance.

Jang et al. [66] used UTE and out of phase (fat and water) MR images which were acquired using dual echo ramped hybrid encoding (dRHE) to segment the brain into three classes: air, soft tissue, and bone. UTE sequences used as an input to retrain a pretrained deep network [86] with T1-weighted MR images. Transfer learning was applied to adopt the knowledge learnt from other MR sequence to UTE sequences to improve the learning and obtain a reliable training. The obtained segmented MR images were processed using conditional random field technique to refine the segmentation results. Furthermore, the out of phase images were used to segment the soft tissue into fat and water components using two-point Dixon-based segmentation. The segmented labels from deep learning- and Dixon-based water and fat images were integrated to generate the pseudo CT images. The proposed method was compared with three other MR-based attenuation correction methods while using CT-based attenuation correction map as the standard reference. Dice similarity score between the predicted labels and CT ground truth images was calculated to evaluate the segmentation results. The corrected PET images were evaluated using relative PET errors. The results showed that this method is clinically feasible with rapid dRHE acquisition time and less than 1% relative PET error in most brain regions. They also showed that the application of conditional random field did not increase the computation time but improved the air and bone detection as well as it corrected some artifacts.

Arabi et al. [84] proposed a deep learning generative adversarial semantic model that generates pseudo CT images for MR image-based attenuation correction. The generative adversarial network consists of two main components: synthesis network and segmentation network. The synthesis part generates pseudo CT images from T1-weighted MR images and the segmentation network segments the obtained pseudo CT images into four tissue classes which are bone, air, soft tissue, and background. The two blocks are connected to each other since the segmentation network contributes to the backpropagation process on the synthesis network. The method was compared with an atlas-based method and a commercial segmentation based method by calculating the cortical bone dice similarity coefficient, mean error, mean absolute error, SUV error, relative mean square error (RMSE), PSNR, and structural similarity index measurement (SSIM). They found the proposed method and atlas-based method have similar performance with tolerable errors. They also concluded that the deep learning method outperforms the commercial segmentation-based approach used in the clinic. CT-based attenuation correction maps were used as standard reference for evaluation.

## Performance Metrics and Clinical Evaluation

### Performance Metrics

The segmentation accuracy is crucial for medical image analysis and quantification. Usually, the segmentation is evaluated using different evaluation metrics such as dice similarity coefficient or F1- measure [63], Jaccard index [87], sensitivity (recall) [62], specificity (precision) [62], accuracy [64], AUC-ROC [64], and false discovery rate [4].

The attenuation correction map is usually evaluated by calculating the PET reconstruction error [56], MAPE [58], RMSE [57], relative difference maps [63], and visual comparison of the maps [61]. The majority of studies used CT attenuation correction map as the gold standard reference for validation [52–58].

### Clinical Evaluation of PET Attenuation Correction Maps

A variety of clinical studies were carried out to evaluate the clinical performance of MR image-based PET attenuation correction methods. Tables 4 and 5 summarize the clinical studies with their evaluation metrics.

**Table 4:** Clinical evaluation studies of MR image-based attenuation correction (MRAC) for brain PET images

Reference	Attenuation correction method	MR sequence	Clinical case	Ground truth	Evaluation metrics
[88]	Siemens MRAC (version VB20P)	UTE	Cancer	CT-based attenuation correction	Visual assessment Dice = 0.65 Relative difference in the entire head = 29% Relative difference in mean SUV range= − 5.2–3.6% Activity concentration overestimation = 0.5–3.6 % Activity concentration underestimation = 2.7–5.2%
[89]	MRAC with bone segmentation	UTE	Parkinsonism	CT-based attenuation correction	Visual assessment Mean difference of binding ratio = 0.66 Intraclass correlation coefficients for putamen = 0.967 Intraclass correlation coefficients for caudate nucleus = 0.682
[90]	MR-based attenuation correction by applying bone segmentation	Proton density-weighted ZTE	Cancer	CT-based attenuation correction	Average Jaccard distance = 52% ± 6% Qualitative scoring of by an experienced radiologist and nuclear medicine physician = 1.7 ± 0.5–0.3 ± 0.6
[91]	MRAC by applying segmentation and assigning continuous attenuation values to the bone	ZTE	Cancer	CT-based attenuation correction	Visual assessment Relative difference in the temporal lobe = 2.46% ± 1.19% Relative difference in the cerebellum = 3.31% ± 1.70% Absolute relative difference (all volume of interests) = 1.77% ± 1.41
[92]	MRAC with bone segmentation	T1 weighted	Cancer	Dixon-based attenuation correction without bone segmentation	Visual assessment Relative difference = 5–20 % Mean relative correlation coefficient between relative perfusion and relative glucose uptake = 0.53
[93]	MRAC method with Dixon water-fat segmentation	T1 weighted	Dementia	CT-based attenuation correction map	Visual assessment Underestimation of 25% in the cortical regions and 5–10% in the central regions of the brain

**Table 5:** Clinical evaluation studies of MR image-based attenuation correction (MRAC) for brain PET images (Continued)

Reference	Attenuation correction method	MR sequence	Clinical case	Ground truth	Evaluation metrics
[94]	MRAC with Dixon and UTE	Dixon and UTE	Epileptogenic and dementia	CT-based attenuation correction	Activity concentration difference (Dixon − cortical gray matter) = 21.3% Activity concentration difference (UTE − cortical gray matter) = 15.7% Activity concentration difference (Dixon − cerebellum) = 19.8 % Activity concentration difference (UTE − cerebellum) = 17.3 % Differences in regional SUV ratio (UTE) = between − 0.77 ± 0.26 and 14.27 ± 0.94
[95]	MRAC with segmentation using gaussian mixture model with two methods of attenuation coefficients assignments (constant continuous values)	UTE	Healthy	Manually segmented MR images and CT-based attenuation correction	Visual assessment Dice (Air) = 0.985 ± 0.02 Dice (Bone) = 0.737 ± 0.17 FP (Air) = 0.007 ± 0.07 FP (Bone) = 0.215 ± 0.84 FN (Air) = 0.022 ± 0.09 FN (Bone) = 0.277 ± 0.98 Relative error-fix (Full brain) = 0.0 ± 2.0 Relative error-continous (Full brain) = 1.3 ± 1.9 Voxel-wise difference map Brain regions histogram

For example, Aasheim et al. [88] evaluated the performance of the most recent version of Siemens UTE-based attenuation correction for PET data using seven lymphoma and twelve lung cancer patients. They concluded that further improvement is needed for accurate segmentation of bone. Choi et al. [89] studied the clinical quantification of PET using UTE-based attenuation correction including bone segmentation. They found that UTE-based attenuation correction causes spatial bias in PET quantification.

Delso et al. [90] were the first to publish a clinical evaluation of bone identification based on brain ZTE sequences. They reviewed the attenuation maps from 15 clinical datasets acquired with a PET/CT/MR trimodality setup and they found out that ZTE images are an efficient imaging sequence to overcome the limitation of bone tissue in attenuation correction maps with sufficient accuracy. Moreover, the evaluation results showed that ZTE sequence is better than UTE according to Jaccard distance value. Sekine et al. [91] proposed a study to evaluate the clinical feasibility of ZTE-based attenuation correction compared with a clinical applied method based on atlas attenuation correction. The calculations showed that the absolute relative difference between PET images is improved with ZTE-based attenuation correction. They concluded that this method is more accurate than clinical atlas attenuation correction.

Anazodo et al. [92] evaluated the addition of bone information on Dixon attenuation correction maps from T1-weighted MR images and the results proved the improvement of underestimation of PET activity. Andersen et al. [93] assessed the regional and absolute bias introduced from neglecting bone using different Dixon image-based methods. They concluded that further improvement for the existing methods is required to adopt PET/MR imaging in clinical routine.

Dickson et al. [94] assessed the quantitative accuracy of Dixon- and UTE-based MR image attenuation correction methods which were compared with CT-based attenuation correction method. Significant underestimations of activity concentrations were found using both Dixon and UTE sequences. The underestimation using UTE is less than with Dixon attenuation correction.

Baran et al. [95] developed a segmentation-based method which compared with three other MR-based methods. The proposed method is based on gaussian mixture segmentation with two different approaches of attenuation coefficients assignments (constant and continuous values). This study firstly compared two attenuation coefficients reference maps using manually segmented MR image- and CT-based maps and concluded that there is a very small mean differences across all subjects. Then, the proposed method was evaluated with CT-based attenuation coefficients and compared with the vendor UTE and UCL methods [68]. They found out that the reconstructed PET with continuous attenuation coefficients has a better agreement with the reference map especially in the cortical bones region while UCL method showed an overestimation for all brain regions. They also observed that the significant differences appear in the cerebellum region. Moreover, the segmentation results showed an underestimation in the esophagus region.

Finally, Ladefoged et al. [96] published a comparison study that evaluates eleven selected MR attenuation correction methods from the literature to study their feasibility in the clinical domain. The evaluated methods are two vendor-implemented using Dixon and UTE sequences, five atlas-based methods, one emission-based method, and three segmentation-based methods. They used a large dataset which contains 359 patients. The main finding of this study is all methods do not exceed more than 5% relative error in the whole brain and in all brain regions compared with CT-based reference map. They also found out that three template-based methods [68, 70, 97] and one segmentation-based method [50] outperform the others in terms of robustness, outliers, and clinical feasibility. Another conclusion is the vendor-implemented methods outperform other methods in term of processing time. The main limitation of this study is the results cannot be generalized since the subject datasets do not include children and patients with anatomical changes.

## Challenges and Future Opportunities

### MR Image Segmentation: Challenges and Opportunities

Although MR images can provide needed information for PET attenuation correction by the aforementioned methods such as tissue segmentation, MR image segmentation itself is challenging. A common problem with segmentation techniques is the misclassification of the pixels which makes the determination of boundaries very challenging. This may lead to false negatives where the lesion regions classified as healthy and false positive where the healthy regions identified as diseased lesions. Hence, robust and accurate segmentation techniques are required to be used in clinical routine.

Machine learning techniques such as neural networks, clustering, random forest, and SVM presented good accuracy with different sets of features. The combination of different techniques for different medical applications leads to better performance and accuracy such as combining random forest classifier with Markov random field to segment white matter lesions in contrast-enhanced FLAIR MR images [29] and combining regularized SVM with a kNN classifier for hippocampus segmentation as represented in [98].

Comparison between the performance and efficiency of each technique is generally applied within the same context and same medical application. For instance, there are several studies that compared the segmentation results using different classifiers as represented in [19, 99–103]. The comparison study between different types of classifiers showed the superiority of deep neural networks and especially CNN in the segmentation task.

The conventional machine learning workflow composes of features extraction and selection then classification. However, the feature vectors control the performance of segmentation methods rather than the classifier’s type. Therefore, more attention on the development of the feature extraction techniques should be carried out with the usage of simple classifiers. Deep learning is the best solution to avoid the hand-crafted features as the training process of the deep network learns the features automatically through the convolutional layers. At the end of the learning process, only high-level features that represent the main characteristics of the data are preserved.

Although deep learning has shown to be superior than any other machine learning techniques, it needs a good estimation of the numerous parameters of the network as well as it needs excessive training time to obtain the weights which will be used for predictions.

### MR Image-Based Attenuation Correction: Challenges and Opportunities

Hybrid PET/MR scanners introduced the complementary nature of MR and PET images to clinical applications and have improved the PET quantification for disease diagnosis and treatment planning by providing different tissue characteristics. Furthermore, it also reduced the acquisition time in case of simultaneous acquisitions. The mergence of MR image modality into the field of PET attenuation correction and quantification raised the new challenges and difficulties.

Table 2 summarizes two types of segmentation methods which are applied on PET attenuation correction: supervised and unsupervised techniques. Fuzzy c-means clustering is the only unsupervised technique which is applied in the literature. However, most of these studies [8, 62] are evaluating the segmentation results by reporting the accuracy values which leads to an inaccurate evaluation since the accuracy metric is not applicable in the case of class unbalancing issue. The high values of accuracy do not indicate a robust segmentation result. On the other hand, the supervised machine learning techniques are as follows: SVM, random forest, and adaptive boosting classifiers. The random forest-based approach [64] used PET features combined with other MR image features and achieved the dice value 0.98. This method is not comparable with other methods that rely on MR features only. Moreover, the reviewed methods used different MR sequences; hence, there is no way to compare the results. Some studies used simple and conventional MR sequences such as T1-w [63, 65, 73] and others used more sophisticated sequences such as UTE sequences [61, 66].

The main limitation of deep learning-based method is the availability of big data especially with medical datasets. The deep models require a lot of data to be well trained and tested. Sufficient training data with different abnormalities should build deep learning models that can perform better than atlas-based methods which assume healthy tissue of each patient. Furthermore, the computation time of large datasets is another challenge of training deep models. The training process can take several days; hence, the usage of high performance computers and graphical processing units is mandatory. The promising thing is once the model is trained, the predication time is very short. Another common limitation in the studies that applied deep learning for PET attenuation correction is the need to apply co-registration between MR and CT images. The mis-registration can lead to further errors in the segmentation and PET reconstructions processes. Moreover, the loss function that calculates the training error can cause issues especially in the case of data unbalancing where some classes are minorities such as the bone class in the brain. Therefore, the prediction will be biased toward the majority classes and cause high specificity and low sensitivity segmentation. Voxel-wise methods [104], class weighting [18] and customized loss functions [105, 106] are some potential solutions.

MR image-based segmentation methods are the clinically adopted method in commercial scanners for attenuation correction [47]. These methods are easy to implement with low computational cost. However, they suffer from a poor segmentation by misclassifying bone (by air or soft tissue) due to a low T2 relaxation time. Consequently, the lack of good bone segmentation leads to inaccurate attenuation coefficient map which produces a strong spatial bias of the PET activity. For instance, ignoring the bone attenuation coefficients in the head can lead to 20% underestimation of PET activity [107].

Another challenge is the assignment of attenuation coefficients to each tissue type. Currently, there is no agreement on the value for each class where. For instance, soft tissue linear attenuation coefficient ranges between 0.094 and 0.100, while trabecular bone has one single value assigned to 0.110 and cortical bone ranges from 0.120 to 0.172 [47]. This variation of attenuation coefficients effects the accuracy of attenuation correction map even if the segmentation accuracy is high.

One more important limitation is the assignment of discrete attenuation coefficients while the density of body tissue is represented by continuous values. There is a need to explore and develop segmentation methods that measure continuous attenuation coefficient values for bone and other tissues in order to obtain accurate PET quantification.

Moreover, there is an interpatient variability of tissue attenuation coefficient based on gender and age. This variability can cause non-negligible errors especially in the tissue regions that show high interpatient variability such as bone.

Additionally, there are other, clinically related, challenges and difficulties that affect MR image-based attenuation correction which are outside the scope of this review such as body truncation artifacts, the presence of ancillary objects during the scanning such as the patient bed, MR coils, positioning aids, and medical probes [108].

### Emerging Techniques

Deep learning-based methods were proposed for pelvic and prostate PET attenuation correction. For instance, Bradshaw et al. [109] applied a 3D CNN called DeepMedic [110] to segment pelvis T1- and T2-weighted MR images for PET attenuation correction. Beside segmentation-based methods, there are other works that applied deep learning for attenuation correction for brain [111] and pelvic [112] to learn the relationship between MR and CT images then generate pseudo CT images. Deep learning showed its superiority to classical techniques for MR images segmentation. However, the applications of deep learning for brain MR image segmentation for attenuation correction are limited.

There are other studies that utilized deep learning-based methods for MR image-guided radiation therapy and treatment planning for brain tumor [113–115], prostate/pelvic region [116] and other different whole-body tissues [117]. These studies applied different deep models such as standard CNN, dilated CNN, and generative adversarial networks (GAN). A comparison study [113] between one segmentation, four atlas, and one deep learning methods to evaluate the MR image-based radiotherapy planning in the pelvic region study showed the outperformance of deep learning method in terms of segmentation accuracy, CT generation accuracy, and dosimetric evaluation.

In terms of MR sequences, currently there is room for improvement of the PET attenuation correction performance by using more sophisticated MR sequences with high signal intensity for bone such as UTE, ZTE, or Dixon sequences. These sequences are able to capture very short T2 values [57, 60] which enables accurate detection of bone tissue and, in turn, improves the attenuation correction map. Another elegant way is acquiring UTE sequences at different echo time to extract more information. Moreover, Dixon sequences provides easy access to four tissue classes which are soft tissue, air, fat, and lung.

The usage of the special sequences such as UTE, ZTE, and Dixon images along with robust segmentation techniques and continuous linear attenuation coefficients can achieve better accuracy for PET attenuation correction than atlas-based methods. However, the major drawback of this sequences in the long acquisition time potentially hampering clinical flow.

## Conclusion

This article presented the application of machine learning techniques for MR image segmentation-based PET attenuation correction in hybrid PET/MR scanners. Among machine learning techniques, clustering, classification, and deep learning are proposed in the literature for tissue segmentation. Deep learning approach outperforms other classical machine learning techniques in this task. Although much progress has been made recently with machine learning methods for segmentation, the reported deep learning methods are fewer especially for brain PET attenuation correction. In summary, deep learning is needed to improve the segmentation and attenuation correction accuracy and intensive clinical evaluation studies of deep learning approach are required.
